# Voltammmetric Determination of Captopril Using Multiwall Carbon Nanotubes Paste Electrode in the Presence of Isoproterenol as a Mediator

**Published:** 2016

**Authors:** Sadeghi Akbari chermini, Hasan Krimi, Mohsen Keyvanfard, Khadijeh Alizad

**Affiliations:** a *Young Researchers and Elite Club, Majlesi Branch, Islamic Azad University, Isfahan, Iran. *; b *Department of Chemistry, Majlesi Branch, Islamic Azad University, Isfahan, Iran.*

**Keywords:** Captopril, Isoproterenol, Multiwall carbon nanotubes, Sensor, Voltammetry

## Abstract

The electrocatalytic oxidation of captopril (CAP) was studied by modified carbon nanotubes paste electrode in the presence of isoproterenol (ISPT) using cyclic voltammetry (CV), chronoamperometry and square wave voltammetry (SWV). Also, the values of catalytic rate constant (k), and electron transfer coefficient (α) for CAP were calculated. The mechanism of CA electrochemical behavior at the modified electrode surface was analyzed by various electrochemical methods in the presence of mediator. The prepared modified electrode showed voltammetric responses with high sensitivity for CAP, making it very suitable for the detection of CAP at trace levels. Under the optimized conditions, the peak current was linear to CAP concentration over the concentration range of 0.3 to 90 μmol L^−1^ using SWV. The detection limit was 0.1 μmol L^−1^. The proposed method was successfully applied for the determination of CAP in the urine, tablet and patient urine samples.

## Introduction

Electrochemical based techniques using modified electrodes can be considered for the determination of environmental, biological and pharmaceutical compounds as strong alternatives to the other instrumental methods (1-5). The chemically modified electrodes (CMEs) are very interesting and powerful tools for the analysis of many substances at trace level, using very sensitive electroanalytical techniques (6-9). A significant point in CMEs utilization in speciation work is to choose the most convenient modifier for each analyte, because the sensitivity and selectivity of the electroanalytical response depend on the characteristics of the modifier (10-13).

Since the ’rediscovery’ of CNTs by Iijima in 1991, electrochemical sensing based on carbon nanotubes (CNTs) has grown into a fully fledged research field (14). The extraordinary electrochemical features of CNTs make them suitable for use in Faradaic processes. CNTs modified electrodes have many advantages over other forms of carbon electrodes due to their small size, high electrical and thermal conductivity, high chemical stability, high mechanical strength and high specific surface area which make them very promising candidates in a wide range of applications (15-19).

**Figure 1:**
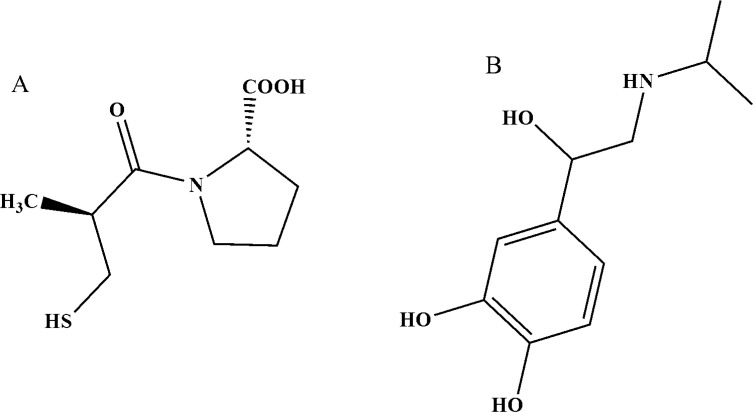
A) Structure of captopril and B) Structure of isoproterenol

Captopril (Figure 1A) is an antiotebsin-converting enzyme inhibitor (ACE inhibitor) used for the treatment of hypertension and some types of congestive diseases. It was the first ACE inhibitor developed and was considered a breakthrough due to its novel mechanism of action and for the revolutionary development process (20). Captopril is a unique antihypertensive drug as it is the only one with a thoil-group in its structure. This gives it the ability to act as a scavenger of free radicals in living systems. A further advantage of the pharmaceutical is its antioxidant properties (21). The determination of captopril is important both from a physiological point of view and for quality control purposes. 

In this study, we described initially the application of ISPT (Figure 1B) as a suitable mediator in the electrocatalysis and voltammetric determination of CAP in an aqueous buffer solution. In continuous, in order to demonstrate the catalytic ability of the modified electrode in the electrooxidation of CAP in real samples, the method was employed for the voltammetric determination of CAP in urine samples from both patients and healthy subjects on the CAP and tablet sample. Table 1 shows a comparison of the figures of merit of the proposed method with those of recently published voltammetric methods for the determination of CAP. As shown, the selectivity and sensitivity of the proposed method is comparing with other publication papers. On the other hand, application and preparation of this modified electrode is easy.

## Experimental

*Reagents and apparatus*

All chemicals used were of analytical reagent grade and were purchased from Merck (Darmstadt, Germany) unless otherwise stated. Doubly distilled water was used throughout. 

A 1.0 × 10^–3^ mol L^–1^ captopril solution was prepared daily by dissolving 0.022 g captopril in water and the solution was diluted to 100 mL with water in a 100-mL volumetric flask. The solution was kept in a refrigerator at 4 ^o^C in dark. More dilute solutions were prepared by serial dilution with water. Phosphate buffer (sodium dihydrogen phosphate and disodum monohydrogen phophate plus sodium hydroxide, 0.1 mol L^–1^) solutions with different pH values were used. 

**Table 1:** Comparison of figures of merit of the proposed method with recently published voltammetric methods for the determination of CAP

**Electrode**	**Method**	**LOD** a **μmol L−1**	**LDR** b **μmol L−1**	**Catalytic** **Potential mV**	**Sensitivity/** **µA µmol** ^-1^ ** L**	**Ref.**
GC c	DPV d	4.8	8-1000	625	14.272	(22)
CPE e	SWV f	0.08	0.2-400	430	0.1091	(23)
CPE	DPV	0.007	0.2-800	220	0.216	(24)
CPE	LSV^ g^	0.009	0.3-300	750	0.1108	(25)
CPE	DPV	0.87	1.0-430	≈380	0.156	(26)
CPE	SWV	0.1	0.3-90	525	0.0252	This work

a LOD Limit of detection;

b LDR Linear dynamic range;

c Glassy carbon electrode;

d Differential pulse voltammetry;

e Carbon paste electrode;

f Square wave voltammetry;

g Linear sweep voltammetry

Spectrally pure graphite powder (particle size <50 µm) from Merck and multiwall carbon nanotubes (> 90%, MWCNTs, d × l = (90 – 70 nm) × (5 – 9 μm)) from Fluka were used as the substrate for the preparation of the carbon paste electrode. High viscosity paraffin (d = 0.88 kg L^–1^) from Merck was used as the pasting liquid for the preparation of the paste electrodes.

All the voltammetric measurements were performed using an Autolab PGSTAT 302N, potentiostat/galvanostat (Utrecht, The Netherlands) connected to a three-electrode cell, Metrohm (Herisau, Switzerland) Model 663 VA stand, linked with a computer (Pentium IV, 1,200 MHz) and with Autolab software. A platinum wire was used as the auxiliary electrode. Multiwall carbon nanotubes paste electrode (MWCNTPE) and Ag/AgCl/KCl_sat_ were used as the working and reference electrodes, respectively. A digital pH/mV-meter (Metrohm model 710) was applied for pH measurements.

*Preparation of the electrode*

To eliminate any metal oxide catalysts within the nanotubes, multiwall carbon nanotubes were refluxed in the 2.0 M HNO_3_ for 12 h, and then washed with twice-distilled water and dried at room temperature. To obtain the best conditions in the preparation of the modified electrode, we optimized the ratio of MWCNTs. The results showed that the better CV shape and current were achieved with 10.0% (w/w) MWCNTs and 90.0% (w/w) graphite. 

According to above points, graphite powder (0.900 g) was dissolved in diethyl ether and hand mixed with 0.100 g carbon nanotubes in a mortar and pestle. The solvent was evaporated by stirring. A syringe was used to add paraffin to the mixture, which was mixed well for 50 min until a uniformly wetted paste, was obtained. The paste was then packed into a glass tube. Electrical contact was made by pushing a copper wire down the glass tube into the back of the mixture. When necessary, a new surface was obtained by pushing an excess of the paste out of the tube and polishing it on a weighing paper.

*Preparation of real samples*

The tablet solution was prepared by completely grinding and homogenizing ten tablets of captopril, labeled 25 and/or 50 mg per tablet. Then, 10 mg of each tablet powder was accurately weighed and dissolved in 100 mL water by ultrasonication. After mixing completely, the mixture was filtered on an ordinary filter paper, 10 mL of which was subsequently transferred into a 100–mL volumetric flask and diluted to the mark with water. Then, 1.0 mL of the solution plus 4.5 mL of the buffer (pH 4.0) was used for analysis using the standard addition method.

The urine samples were stored in a refrigerator immediately after collection. Ten milliliters of the sample was centrifuged for 15 min at 1500 rpm. The supernatant was filtered using a 0.45 µm filter and then diluted 5–times with PBS (pH 4.0). The solution was transferred into the voltammetric cell to be analyzed without any further pretreatment. 

*Optimization of ISPT concentration*

The influence of ISPT concentration on the electrocatalytic oxidation peak current was studied at three different concentrations of CAP at pH 4.0, and in the range of 100.0 to 700 µmol L^-1^ ISPT. The results showed that by increasing the concentration of ISPT up to 500 µmol L^-1^ the peak current increased, whereas higher concentrations of ISPT caused a slight decrease on the magnitude of peak current, which may be due to the formation of ISPT aggregates. Therefore, 500 µmol L^-1^ ISPT concentrations were selected for further studies.

## Results and discussion

*Electrochemistry of ISPT*

**Figure 2:**
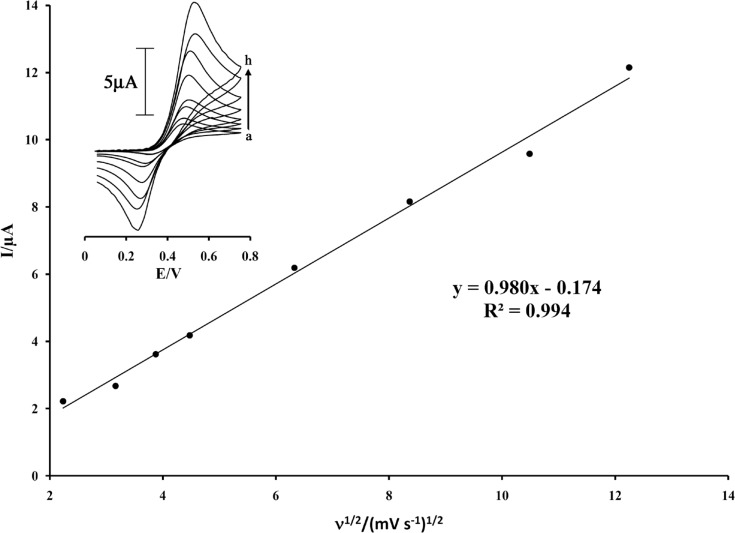
Plot of I_pa_ versus ν^1/2 ^for the oxidation of 200 µmol L^-1^ ISPT at a surface of MWCNTPE. Insert cyclic voltammograms of at various scan rates: (a) 5; (b) 10; (c) 15; (d) 20; (e) 40; (f) 70; (g) 110; and (h) 150 mV s^–1^ in 0.1 mol L^-1^ PBS (pH 4.0

The electrochemical behavior of the ISPT was characterized by cyclic voltammetry. Figure 2 (inset) shows the cyclic voltammograms of ISPT at MWCNTPE in the PBS (pH 4.0) at various scan rates. The experimental results showed well defined and reproducible anodic and cathodic peaks related to ISPT_(red)_/ ISPT_(ox)_ redox coupled with a quasi reversible behavior and with a peak separation potential of ΔE_p_(E_pa_−E_pc_)=205 mV. These cyclic voltammograms were used to examine the variation of the peak currents vs. the square root of potential scan rates. The plot of the anodic peak current was linearly dependent on ν^1/2^ with a correlation coefficient of 0.994 at all scan rates (Figure 2). 

The active surface areas of the modified electrodes are estimated according to the slope of the I_p_ vs. ν^1/2^ plot for a known concentration of K_4_Fe(CN)_6_, based on the Randles–Sevcik equation:

 (1) I_p_ = 269000n^3/2^AD_R_^1/2 ^ν^1/2^ C_0 _

where I_pa_ refers to the anodic peak current, n the electron transfer number, A the surface area of the electrode, D_R_ the diffusion coefficient, C_0_ the concentration of K_4_Fe(CN)_6_ and ν is the scan rate. For 1.0 mmol L^−1^ K_4_Fe(CN)_6_ in 0.10 mol L^−1^ KCl electrolyte with n=1 and D_R_ = 7.6×10^−6^ cm^2^ s^−1^ and from the slope of the I_pa_–ν^1/2 ^relation, the microscopic areas were calculated. The active surface areas were equal to 0.09 and 0.18 cm^2^ for carbon paste electrode (CPE) and MWCNTPE, respectively. The result shows that the presences of MWCNTPE cause increasing the active surface of the electrode.

*Catalytic effect*

**Figure 3:**
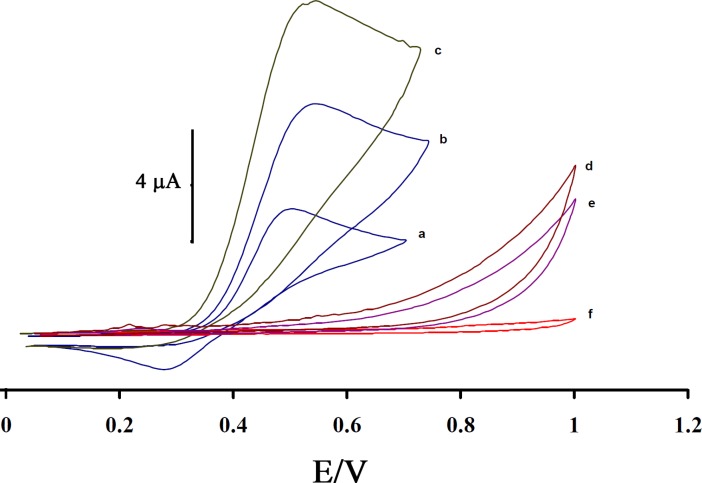
Cyclic voltammograms of (a) the 200 µmol L^-1^ ISPT at the surface of MWCNTPE in 0.1 mol L^-1^ PBS (pH 4.0); (b) 200 µmol L^-1^ ISPT +100 µmol L^-1^ CAP at the surface of carbon paste electrode; (c) 200 µmol L^-1^ ISPT +100 µmol L^-1^ CAP at the surface of MWCNTPE; (d) 100 µmol L^-1^ CAP at the surface of MWCNTPE; (e) 100 µmol L^-1^ CAP at the surface of carbon paste electrode, (f) For the buffer solution at the surface of unmodified electrode (carbon paste electrode); scan rate of 20 mV s^-^

Figure 3 shows the electrocatalytic oxidation of CAP in the absence or presence of ISPT at a MWCNTPE surface. As is obvious, at the potential range studied (0.05–1.05 V), CAP was not electroactive in the absence of mediator at a surface of MWCNTPE and CPE (Figure 3(e and f)), respectively. On the other hand, the anodic current of ISPT was increased substantially in the presence of low concentrations of CAP at a surface of MWCNTPE and CPE (Figures 3(c) and 3b)), respectively. This observation is an evidence for electrocatalytic oxidation of CAP by ISPT. Similarly, when we compared the oxidation of CAP at the surface of MWCNTPE (curve c) and at CPE (curve b) in the presence of mediator, a dramatic enhancement was observed in the anodic peak current at MWCNTPE vs. the value obtained with CPE. In other words, the data obtained clearly show that the combination of MWNTs and the mediator definitely improve the characteristics of the electrode for the oxidation of CAP. The ISPT at a surface of MWCNTPE, in 0.1 mol L^-1^ PBS (pH 4.0) and without CAP in solution, exhibited a well-behaved redox reaction (curve a). The process corresponds to an EC’ (catalytic) mechanism (see scheme 1) (27-30), where the electrochemically formed ISPT_(Ox)_ reacts chemically with CAP diffused toward the electrode surface, while the simultaneous oxidation of the regenerated ISPT_(Red)_ causes an increase in the anodic current. For the same reason, the cathodic current of the modified electrode is smaller in the presence of CAP. 

Since CAP has a thiol moiety, we anticipated that the oxidation of CAP would be pH dependent. In order to ascertain this, the voltammetric response of CAP at a surface of MWCNTPE in the presence mediator was obtained in solutions with varying pH. Result shows that the maximum value of the peak current was appeared at pH 4.0, so this value was selected throughout the experiments (Not shown).

**Figure 4:**
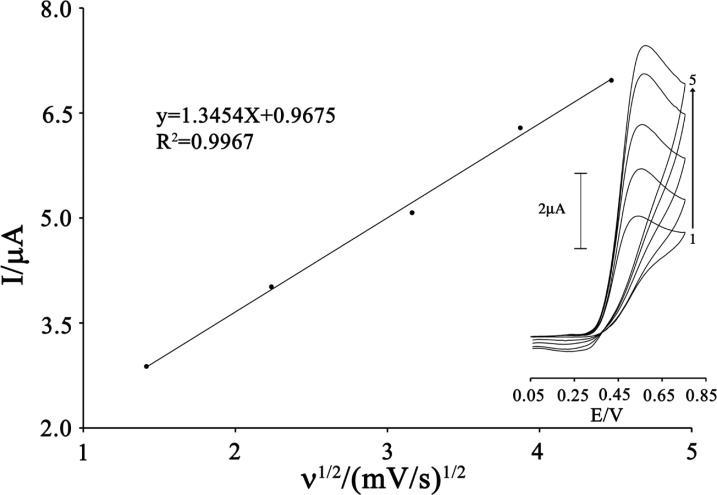
Plot of I_pa_ versus ν^1/2^ for the oxidation of 80 µmol L^-1^ CAP in the presence 200 µmol L^-1^ ISPT at the surface of MWCNTPE. Inset) Cyclic voltammograms of 80 µmol L^-1^ CAP in the presence 200 µmol L^-1^ ISPT at various scan rates as (a) 2, (b) 5, (c) 10; (d) 15 and e) 20 mV s^−1^ in 0.1 mol L^-1^ buffer solution (pH 4.0

The effects of scan rate (υ) on the oxidation current of CAP were also examined (Figure 4). The peak current increased linearly with the increasing the square root of scan rate that ranged from 2 to 20 mV s^–1^ and it can be expressed as follows: 

 (2) I_p_=1.3454 ν^1/2^+ 0.9675 (r^2^=0.9967, I in µA, ν in mV s^–1^)

This result shows that the electrode process is controlled under the diffusion step.

**Figure 5:**
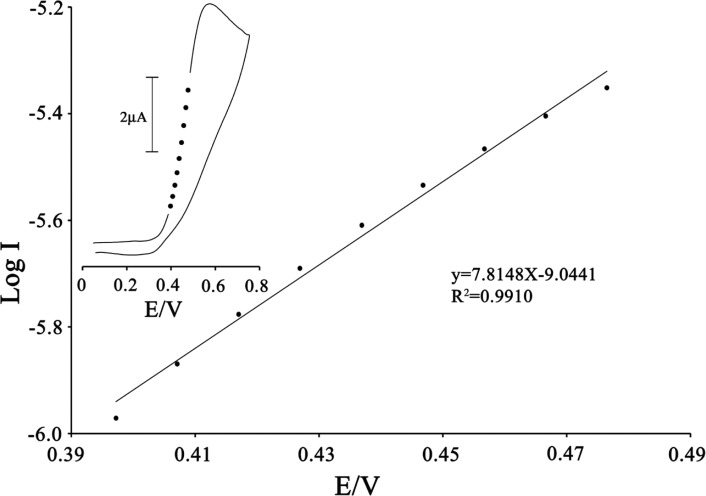
Tafel plot 200 µmol L^-1^ ISPT at the surface of MWCNTPE in 0.1 mol L^-1^ PBS (pH 4.0) at a scan rate of 20 mV s^−1^ in the presence of 100 µmol L^-1^ CAP

To obtain information about the rate-determining step, the Tafel plot was drawn, as derived from points in the Tafel region of the cyclic voltammogram (Figure 5). 

The slope of the Tafel plot was equal to n (1−α) F/2.3RT, which came up to 7.8148 V^-1^ decade. Therefore, we obtained the value of α equal to 0.54.

**Figure 6:**
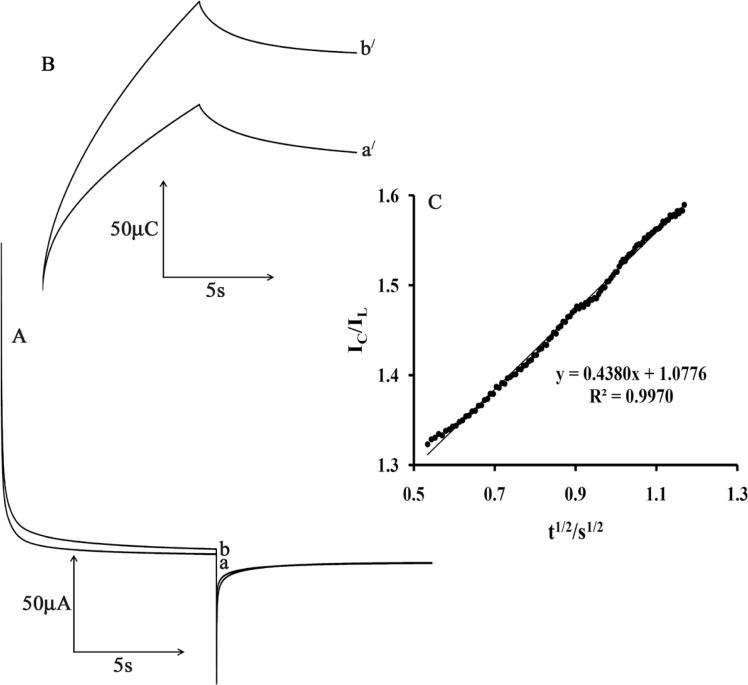
A) Chronoamperograms obtained at the MWCNTPE in the absence a) and in the presence of b) 200 µmol L^-1^ CAP in a buffer solution (pH 4.0). B) The charge-time curves a') for curve (a); and b') for curve (b). C) Dependence of I_c_/I_L_ on the t^1/2 ^derived from the chronoamperogram data

Figure 6A shows the current–time curves of MWCNTPE in the presence of mediator by setting the electrode potential at 0.2 mV (first step) and 70 mV (second step) for different CAP concentrations. As can be seen, there is no net anodic current corresponding to the oxidation of the mediator in the presence of CAP. 

On the other hand, the forward and backward potential step chronoamperometry for the mediator in the absence of CAP shows symmetrical chronoamperogram with an equal charge consumed for the reduction and oxidation of the mediator at the surface of MWCNTPE (Figure 6B, a^/^). On the other hand, the charge value associated with forward chronoamperometry in the presence of CAP is significantly greater than that observed for backward chronoamperometry (Figure 6B, b^/^). 

The rate constant for the chemical reaction between ISPT and CAP (k_h_) is determined according to the method of Galus (31)

 (3) I_C_/I_L_ = π^1/2^ γ^1/2^ = π^1/2^(k_h_t)^1/2^

where I_C_ is the catalytic current of ISPT in the presence of CA and I_L_ is the limiting current in the absence of CA. From the slope of I_C_/I_L_ versus t^1/2^ for five different concentrations of CAP, the average value of k_h_ was calculated to be 3.01 × 10^3^ M^−1^ sec^−1^ (Figure 6C). This value of rate constant explains the sharp catalytic peak observed for the oxidation of CAP at the surface of MWCNTPE in the presence of mediator. 

*Dynamic range and limit of detection*

**Figure 7:**
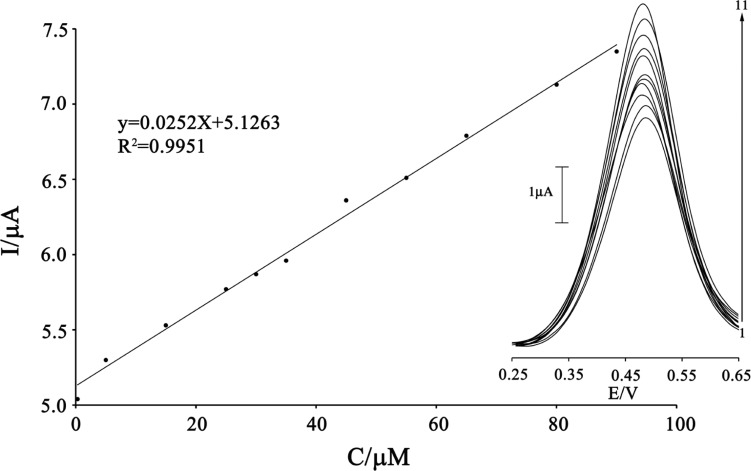
The plots of the electrocatalytic peak current as a function of CAP concentration. Inset shows the SWVs of MWCNTPE in the presence 200 µmol L^-1^ ISPT (pH 4.0) containing different concentrations of CAP. From inner to outer (1–11) correspond to 0.3, 0.5, 15.0, 25.0, 30.0, 35.0, 45.0, 55.0, 65.0, 80.0, and 90.0 μmol L^−1^ of CAP

Square wave voltammetry (with amplitude potential of 50 mV and frequency of 12 Hz) was used to determine the concentration of CAP because SWV, had a much higher current sensitivity and better resolution than cyclic voltammetry, which was used to estimate the lower limit of detection of CAP (Figure 7). Responses were linear with CAP concentrations ranging from 0.3-90 µM and a current sensitivity of 0.0252 µA/(µmol/L). The detection limit was determined at 0.1 µM CAP according to the definition of Y_LOD_=Y_B_+3σ. 

*Interference study*

In order to evaluate the selectivity of the proposed sensor for the determination of CAP, the influence of various foreign species on the determination of 5.0 µmol L^-1^ CAP was investigated. The tolerance limit was taken as the maximum concentration of the foreign substances, which caused an approximately ±5% relative error in the determination. 

The results after the experiments revealed that neither 950–fold of K^+^, Li^+^, Mg^2+^, Br^-^, NO_3_^–^, ClO_4_^–^, SO_4_^2–^,F^–^, glucose, sucrose, lactose, fructose, glycine, urea, histidine, SCN^–^, methionine, alanine, and phenylalanine; nor 700–fold of tryptophan, urea, thiourea, ampicillin and tyrosine; nor 300–fold of uric acid, ascorbic acid, aspirin, hydrochlorothiazide, atenolo, amoxicillin (after addition of 1 mM ascorbic oxidaze) and nor saturation of starch solution affected the selectivity. Also, nor 2–fold glutathione and N-actylcusteine affected the selectivity. Those results confirm the suitable selectivity of the proposed sensor for CAP determination.

*Determination of CA in real samples*

Electrochemical methods have a good sensitivity and selectivity for determination of pharmaceutical and biological sample analysis in real samples.^33-40^ In order to evaluate the applicability of the proposed sensor for the determination of CAP in real samples; we have examined the ability of the electrochemical sensor for the determination of CAP in tablet and urine samples using standard addition method. The samples were also analyzed by a standard method including potentiometric titration with potassium iodate.^32^ The results for the tablet sample analysis are given in Table 2

**Table 2.** Determination of captopril in tablet and urine samples (n=3).

**Sample**	**Captopril added** **(µmol L** ^−1^ **) **	**Expected value** **(µmol L** ^−1^ **)**	**Captopril founded** **(µmol L** ^−1^ **) **	**Standard Method** **(µmol L** ^−1^ **)**
Tablet a	−−	10.0	10.33±0.35	10.55±0.65
	10.0	20.0	19.88±0.45	20.45±0.69
	20.0	40.0	39.75±0.88	39.84±0.75
Tablet b	−−	55.0	55.45±0.55	54.92±0.69
	5.0	60.0	59.65±0.75	60.78±1.01
	10.0	70.0	70.84±0.95	70.95±1.11

a ^50 mg tablet, Darou Pakhsh Company, Iran^

b ^25 mg tablet, Darou Pakhsh Company, Iran^

In addition, the results obtained for the urine samples by the proposed method were compared with the standard method statistically, using Student’s t test (for the accuracy), and variance ratio, F test (for the precision) at 95% confidence level. The results are given in Tables 3. Those results demonstrated the ability of propose sensor for voltammetric determination of CAP in real samples with the good recoveries of the spiked CAP and good reproducibility. 

**Table 3:** Concentration values obtained from the proposed and the reference method for captopril analysis of urine sample using the proposed method under optimum conditions (n=3).

Sample	Proposed method (µmol L^−1^)	Standard method (µmol L^−1^)	*F*_ex_	*F*_tab_	*t*_ex_	*t*_tab(95%)_
Urine^a^	4.14±0.25	4.51±0.65	7.5	19	2.1	3.8
Urine^b^	5.25±0.33	5.62±0.82	8.5	19	2.6	3.8
Urine^c^	7.35±0.75	6.95±0.85	8.7	19	3.8	3.8
Urine^d^	4.55±0.37	4.865±0.55	6.1	19	2.4	3.8

^±^^Shows the standard deviation.^

a ^Sampling was made after 1.5 h from a man who had heart problem and used captopril.^

b ^Sampling was made after 2.0 h from a man who had heart problem and used captopril.^

c ^Sampling was made after 2.5 h from a man who had heart problem and used captopril.^

d ^Sampling was made after 3.0 h from a man who had heart problem and used captopril.^

*Stability and reproducibility*

The repeatability and stability of the MWCNTPE was investigated using square wave voltammetric measurements of 20.0 µmol L^-1^ CAP in the presence of mediator. The relative standard deviation (RSD%) for seven successive assays of CAP was 2.1%. When using six different electrodes, the RSD% for seven measurements of 20.0 µmol L^-1^ CAP was 2.9%. When the modified electrode stored in the laboratory, the response of the modified electrode retained 97% of its initial response value after two week and 92% after 35 days. These results indicate that MWCNTPE has good stability and reproducibility.

## Conclusion

This study demonstrates the construction of a chemically modified carbon paste electrode by incorporation of carbon nanotubes as a suitable electrochemical sensor in the presence of ISPT as a homogeneous mediator for CAP determination at trace level. The new voltammetric sensor for the determination of CAP is very rapid, reproducible, selective and sensitive, and can be used for real sample analysis. The proposed method is a selective, simple and precise method for voltammetric determination of CAP in real samples such as drug and patient urine, as low as 0.1 µmol L^-1^ CAP. In addition, the kinetic parameters of the system have been calculated from the experimental results.
